# Medical Items and News of Interest to the Physician

**Published:** 1883-07

**Authors:** 


### Medical Items and News,

### For the Use of Physicians.

CULLED FROM THE STANDARD MEDICAL JOURNALS OF THE WORLD.

NOTE.—These formulæ are intended only for the regular practitioner of medicine, and should be employed only by him, or under his immediate supervision.

**Pleuritic Pain in Pneumonia.**

Professor Baumler, of the University of Frieburg, applies an ice bag to the chest to relieve the pleuritic pains in pneumonia.

**Hair Restorer.**

The leaves of *eucalyptus* powdered to a pulp, and applied to the head, is recommended as a cure for baldness by a Californian who has tried it.

**Herpes Zoster.**

Painting the vesicles with strong carbolic acid and covering with a layer of thick cotton-wool, has been successfully applied by Dr. Lambuti. He reports a case of the kind in the *Revista Clinica di Bologna.*

**Snake Bites.**

The *Medical Record* says that Prof. Bisoz has found in seventeen cases, that a filtered solution of chloride of lime, injected into the place where the virus entered, prevented any poisonous symptoms appearing.

**Varicose Veins.**

Dr. J. H. Mussen has produced good results in a number of cases of varicose veins from the use of fluid extract of hamamelis in teaspoonful doses. The cases are recorded in the *Medical Times,* April 21, 1883.

**Ovaritis.**

Dr. P. J. Murphy, of Washington, recommends the use of a suppository containing one quarter of a grain of iodoform, combined with a small quantity of the alcoholic extract of belladonna, one of these to be used three times a day per the rectum.

**Basham's Mixture.**

The Canada *Lancet* gives the following formula for the composition of Basham's mixture, so well known in the treatment of albuminuria: Tr. ferri chlor., ℥ss; acid. acet. dil., ℥i; liq. amm. acet., ℥ivss; tr. aurant. cort., ℥iss; glycerine, ℥ss. Sig.—A tablespoonful, largely diluted, three times a day.

**Acne.**

R.

Sulp. præcip., ʒ iv.

Pulv. Camphoræ, gr. x.

Pulv. tragacanth, gr. xx.

Aquæ calcis.

Aquæ rosæ, āā f ℥ ij.

M. S. Apply locally.

**Chordee.**

℞

Kalii bromid., ʒ iss.

Tr. opii., ʒ ss.

Glycerinæ, ℥ ss.

Aquæ distill, ℥ v.

M. S. Four injections daily.

**Diabetes.**

From the *Gaz. degli Ospatali,* we learn that in two cases of diabetes mellitus, Bozzolo has used iodoform (30 grains daily) with good results. In both cases the quantity of urine was decreased; in one (mild) the glycosuria entirely disappeared; in the other (severe), it was lessened.

**Heart Disease.**

Dr. Huchard claims excellent results in the treatment of cardiac dropsy by caffeine. He begins with a dose of 7 grains, gradually increasing to 15, 30, or even 45 grains per diem. He says, however, that such large doses often cause severe pains in the stomach.—*Archives Generales de Medecine.*

**Chronic Diarrhea.**

M. Gubler has found it most useful in the diarrhea of phthisis, and whenever ulceration of the uterus is suspected. He gives it in powders in the following form:

Oxide of zinc, 30 grains.
Bicarbonate of soda, 10 grains.
In four powders, two or three daily.

**Puerperal Eclampsia.**

According to Professor Brens, active diaphoresis will cure puerperal eclampsia, whether occurring in the puerperium, or in the last months of pregnancy. This is induced by the hot bath (40° to 45° C.) followed by the pack. Half an hour is sufficient time for the former, and for the latter two or three hours will suffice. This treatment will also cause œdema and albuminuria to disappear if properly carried out, without interruption of pregnancy. The details of Prof. Brens' treatment are contained in the *Arch. f. Gyn.*

**Boracic Acid as a Surgical Dressing.**

This article has been highly recommended as a surgical dressing dissolved in glycerine, as possessing all the advantages of oily dressings without the disadvantages. Wounds dressed with it can be perfectly cleansed with water, since it is readily miscible with it, while those dressed with oil can not be so washed.

---

**Snake Bite.**

Dr. George H. Carpenter, of Moonfield, W. Va., writes to the *Medical News*, that he has secured excellent results in two cases of poisoning by the bite of the Copperhead, from the internal administration of tinct. iodini comp., fifteen drops in a third of a glass of water, and the local application of the tincture of iodine to the bitten limb or part.

---

**Acute Orchitis.**

Dr. Henderson, of Shanghai, reports, in *The Lancet* of December 16th, three cases of gonorrhœal epididymitis which were rapidly relieved by salicylate of sodium. He advises that it should be used in acute cases only, and in dose never less than a scruple, repeated every hour for at least three doses, after which the interval may be lengthened.—*Med. Record.*

---

**Gouty Pains and Neuralgia.**

Dr. Fronmuller (*Memorabelien*) rubs the solid stick over the moistened surface for about thirty seconds. When dry, the skin is covered with a compress of salicylated cotton, which is removed once or twice a day until the blister is healed. In order to be efficient the irritation should be sufficient to cause an exudation beneath the epidermis.

---

**Contagious Diseases.**

A somewhat singular, and, if substantiated, very valuable observation has been made by Dr. W. Duff Green, of Mt. Vernon, Ill., in the St. Louis *Courier of Medicine*, February, 1883. He relates his experience in six families, wherein he treated typhoid fever or measles. In every individual to whom he had given tincture of the chloride of iron, if the disease appeared at all it was very slight, while the majority of those so treated escaped it altogether; at the same time, nearly all those who did not take the iron had the disease in its ordinary severe form. He has noticed this prophylactic action since 1877, and he only records a few of his many observations. It may be that the iron simply enables the system to withstand the onset of the disease, by giving it tone, without possessing any specific action; but whatever the explanation is, this report is sufficiently valuable and trustworthy to stimulate further investigation.—*Med. and Surg. Reporter.*

---

**Infant Food.**

Dr. S. B. Sherry, of Delafield, Wis., recommends the following formula for a substitute for human milk: Add a pint of barley-water (hot?) to an ounce of *pearl barley;* allow it to cool, and then strain it. Mix one-third of a pint of this barley-water with two-thirds of a pint of fresh undiluted cow's milk, and sweeten with a teaspoonful of *milk-sugar.*—*Am. Journ. of Obstet.*

---

**Coffee in Typhoid Fever.**

The *Medical Record* speaks highly of coffee as a heart and brain stimulant in adynamic conditions, and is of the opinion that perhaps its value is not fully recognized. The recent experiments of Dr. Guillasser, of the French Navy, was spoken of, who has given coffee in the early stages of typhoid fever with marked success. Three teaspoonfuls were given adults every two hours, alternating with one or two teaspoonfuls of Claret or Burgundy wine. A beneficial result was immediately apparent.

---

**Erysipelas of the Face.**

The following rather strong application may be painted over the parts affected once every two hours; a thin layer of cotton being placed over the parts immediately afterwards:

℞

Ac. carbolici.

Sp. vini recti āā ʒ j.

Sp. Terebinth, ʒ j.

Tr. Iodinii, ʒ j.

Glycerine, ʒ v.

M.

---

**Night-Sweats.**

The following powder is recommended by Dr. Konhorn in the night-sweats of phthisis: Acid salicyl. gr. 45, starch, ʒ ijss, chalk, .℥ ijss. The entire body of the patient is dusted with this powder at bedtime. The author claims to have obtained great success by this treatment. The same powder is employed in the Austrian army in sweating of the feet.—*Memorabilien.*

**Diphtheria.**

Dr. G. Murray Gibbes, Taranaki, New Zealand, recommends the use of eucalyptus globulus in the treatment in this affection, and reports thirty-seven cases where the remedy was successfully employed. He keeps the patients in an atmosphere saturated with vapor arising from the leaves placed in boiling water.

**External Hemorrhoids.**

According to the *Drug Cir.*, Dr. Blascho, of Berlin, recommends compresses soaked in a one per cent. solution of ergotine, to be applied hourly. D. Pasqua, of Florence, gives the following ointment as infallible:

Extract of belladonna, gr. v.
Iodoform, acetate of lead āā gr. j.
Petroleum jelly, ℥ j.

Make into an ointment, to be applied three or four times a day.

**Acute Articular Rheumatism.**

F. E. Stewart, M. D., Ph., G., recommends the following formula, which has been used with gratifying success in a number of cases treated by him. Salicylic acid, ʒ iij; divide in three powders and wrap in white paper. Potas. bicarb., ʒ vj; divide in three powders and wrap in blue paper. Give the contents of one white paper and one blue paper, mixed together in a tumbler, with a little water, and repeat three times a day until nine powders are taken. If, at the end of three days, the medicine has produced no marked beneficial effect its further continuance is useless.

[Is not this rather heroic treatment? Sixty grains of salicylic acid at one dose, is three times larger than we should care to indulge in. ED.]

**Diphtheria.**

A mixture of carbolic acid and chloride of lime, under the name of tri-chlorinated phenol, is described by Dianin in the *St. Pet. Med. Woch.* He claims that it is twenty-five times more powerful as a disinfectant than carbolic acid, that in minute proportions it prevents fermentation, that it is a more powerful antiseptic than any other now in use, that it destroys all noxious vapors effectively, while its own odor may be masked by oil of lavender; that while in substance slightly irritating, in solution it is not at all so; that it is of great advantage in the treatment of chancres and diphtheria.—*Chicago Medical Review.*

**Alterative Tonic.**

The following is said to be Dr. Godell's favorite mixture:

℞

Hydrag. bichlor, gr j–ij.

Liq. arsen. chlor., ʒ j

Acidi hydchlor, dil.

Tr. Ferri chlor, aa ʒ ij.

Syr. zingib, ℥ ij.

Aquæ ad, ℥ vj.

M. Sig. Two teaspoonfuls three times daily, in water, after meals.

**Constipation.**

℞
Pulv. aloe, grs. xxx.
Ext. belladonnæ fl, ♏ xx.
Ext. nucis vom. fl, ♏ xxx.
Pulv. ipecac, grs. iij.
Tinct. gentian comp. ℥ ij.
Syr. simp, to make, ℥ iv.

M. Sig.—Teaspoonful on the evening of each day when the bowels have not moved. This dose is for adults. For children, five drops for each year of age.

**Carbolized Iodoform.**

The following formula is given by C. Sherk, (Berliner Klin. Wochenschrift) as a great improvement over iodoform:

℞

Iodoform, x grs.

Acid carbolic, v grs.

Ol. meuth. pip., ij drops.

The acid is to be rubbed up with the iodoform, and the peppermint oil added subsequently. The disagreeable odor of the drug is completely covered, and it is not again developed even at an elevated temperature.

**Stye.**

Dr. Louis Fitzpatrick, who has recently returned from Egypt, where all kinds of eye affections are extremely common, writes to the *Lancet*, that he has never seen a single instance in which the stye continued to develop after the following treatment had been resorted to: The lids should be held apart by the thumb and index finger of the left hand (or a lid retractor, if such be at hand) while the tincture of iodine is painted over the inflamed papilla with a fine camel's hair pencil. The lids should not be allowed to come in contact until the part touched is dry. A few such applications in the twenty-four hours are sufficient.

**Simple Elixir.**

A very pleasant vehicle for the administration of medicines which are to be given in solution is prepared by mixing together two fluid ounces each of orange-flower water and simple syrup, adding half a fluid ounce of alcohol to preserve, and coloring with two drachms of compound tincture of cardamom. This will be found of service to the country physician who is obliged to dispense his own medicine.

**Cancer.**

℞
Sanguinariæ canadensis, grs. xij.
Arsenici iodidi, grs. ij.
Ext. conii, grs. xl.

M. Div. pil. xxiv. S. 1 to be taken three times a day.

℞

Bromidi chlordi, ♏ iij.

Pulv. glycyrrhizæ, grs. lx.

M. Div. in pil. xx. S. 1 pill two or three times daily.—*Med. Gaz.*

**Headache.**

Dr. Haley says, in the *Australian Medical Journal*, that, as a rule, a dull, heavy headache situated over the brows and accompanied by languor, chilliness, and a feeling of general discomfort, with distaste for food, which sometimes approaches to nausea, can be completely removed, in about ten minutes, by a two-grain dose of iodide of potassium dissolved in half a wine glassful of water, this being sipped so that the whole quantity may be consumed in about ten minutes.

**Emmenagogue.**

The *Medical News*, in a recent editorial, directs attention to the emmenagogue properties of the Polygonum Hydropiperoides. As an emmenagogue it is indicated in states of anæmia, functional torpor of the ovaries and uterus due to systemic depression, and is contra-indicated in the condition of plethora. Its power to stimulate the uterine circulation renders it useful in menorrhagia, and in metrorrhagia due to relaxation of the uterine vessels. Subinvolution of the passive kind with a sluggish circulation, cold hands and feet, and general depression, are also benefitted by this remedy. The best form for administration is the fluid extract in 5 to 30 minim doses, mixed with glycerine and wine, 3 or 4 times a day.—*Canadian Practitioner.*

**Asclepias Curassavica.**

Dr. Guim, Aroes, Rio Janeiro (Union Medico), has been investigating the physiological properties of bastard ipecacuanha (asclepias curassavica) and has found that its active principle is a cardiac poison, somewhat similar to digitalis. It is an excitant of the vaso-motor centre. The solutions obtained by maceration of the stalks, and that obtained in like manner from the roots, act with unequal intensity.—*The Druggist.*

**Skin Disease.**

M. Desquin, of Anvers, has given glycerine internally in certain forms of skin disease with, it is said, marked success, especially in acne punctata and the furuncular diathesis. He commences with four drachms daily and gradually increases the dose. He states that the secretion of the cutaneous glands, which is thick and irritating in these diseases, becomes more liquid, and cutaneous irritation is notably lessened. During the convalesence from scarlet fever, he believes that it facilitates desquamation.

**Kava-Kava.**

Kava-Kava, well known to travelers as the intoxicating drink of the Fijians, has lately been introduced as a cure for urethritis and gonorrhœa. From my own observations, I find that its chief action is to reduce the acidity of the urine, and it may be given with benefit in cases where there is an uric acid diathesis. I have found it very useful in enlarged prostate, as it has a very beneficial action on the mucous membrane of the bladder, and prevents any uneasiness which may occur during the temporary retention of the urine.—*British Medical Journal.*

**Vomiting in Pregnancy.**

Pop-corn has been introduced to the materia medica by Dr. F. C. Wallace (*Medical and Surgical Reporter*) as a remedy for the vomiting of pregnancy. It is to be prepared in the usual way in a wire popper and sprinkled lightly with salt, and is to be eaten freely. He speaks from an experience of several cases in which it served a good purpose, and reports one in which accepted remedies had previously failed. Dr. E. J. Kemf, in the *Louisville Medical News*, speaking favorably of personal experience with it, says that Dr. F. A. Burrall called attention to it three years ago.

**Menstrual Flow.**

For the relief of acute pain in the pelvic region, which not infrequently occurs in woman for a day or two antecedent to the occurrence of the menstrual flow, which is no doubt due to hyperæmia and hyperæsthesia of the reproduction organs, the *Virginia Medical Monthly* recommends the following:

℞

Codeiæ sulph, gr. i.

Chloral hydrate, ammonia bromidi āā grs. xv.

Aquæ camphoræ, ℥ j.

M. Sig. For one dose, take at bed time. A repetition of the dose at that time is rarely necessary. In some cases a warm sitz-bath, for fifteen minutes before retiring, may be taken with advantage.

---

**Rheumatism.**

Salicin is the natural product of the willow root, while salicylic acid is the artificial phenol derivative. Dr. Quinlan says that while the latter will sometimes sicken, even in moderate doses, almost any quantity of the former can be given, and in the *Lancet* he records two cases of acute rheumatism rapidly cured by its use. He advises it in large and frequently repeated doses. One of his cases took eighty in one dose, daily, for three days without any bad effect. Salicin, in very large doses, is most easily taken by stirring it into a hock glass of milk, which the patient rapidly swallows before it has time to settle down from its suspension. It may also be taken in wafer paper, or even in water, but in this case the bitter flavor is felt. It should always be taken on an empty stomach; after food it often occasions nausea and tinnitus. Its value in acute rheumatism can not be overestimated, and should the physician get hold of the case early, he will likely be able to conquer it before there is time for cardiac complication, so apt to leave enduring mischief behind it. Finally, when the rheumatic sufferer convalesces the salicine should not be dropped too suddenly. For a week it should be given in full doses morning and evening, and for a fortnight once daily; otherwise a relapse may supervene. A notable feature in the salicine treatment is its tonic effect in promoting a steady and rapid recovery of strength and vigor during convalescence. It is very cheap, selling in England, at retail, for less than twenty-five cents per ounce.

**Epistaxis.**

M. Auquier (*Gaz. hebd. de Montpelier*) mentions a case in which he was called to a man of 20 who had been suffering for three hours from violent epistaxis. The patient had been subject to such attacks from infancy. M. Auquier tried in vain to stop the bleeding by means of cold water, plugging the nares, mustard-plasters, etc. At last he irritated the nose with very hot water, with instant success. During the next night and day the friends of the youth were able by this means to stop at the outset several fresh outbreaks. The author thinks the hot water acted by producing a reflex contraction of the bleeding vessels; and not by encouraging the flow and so causing depletion of the superficial vessels, as has been supposed, in reference to the stoppage of uterine hemorrhage.—*Practitioner.*

---

**Eczema.**

Locally, the following lotion will be employed:

℞.

Acidi carbolici, ♏xl.

Alcoholis, f ʒ v.

Glycerinæ, fʒij.

Zinci oxidi,

Amyli, āā ʒ ij.

Aquam, ad. f ℥ viij.

Misce.

This is to be dabbed lightly on the parts three times daily, allowing the sediment to dry and remain there. In four or five days the local treatment will probably be changed, since by that time the disease will doubtless have changed its form. A mild calomel ointment, a scruple to the ounce, may then prove useful.—*Prof. L. A. Duhring.*

---

**Chloral Hydrate as a Vesicant.**

The vesicating power of this drug was discovered by Dr. F. D. Ritter, of Gaines, Pa. It is applied by sprinkling upon ordinary adhesive plaster, melted by a gentle heat, and applied while warm to the part to be vesicated. In three minutes a gentle heat is felt, "increasing in intensity for about three minutes more till it is like to burn, then gradually easing off, until at the end of ten minutes the parts feel free from pain. The secondary effect is soothing. In some instances within a half an hour a second burning is felt, though not so intense as at first, nor so lasting." If, at the end of ten minutes the plaster is removed, a blister will be found, the surface being as effectually denuded in this brief time as a cantharidæ plaster would have done after six hours' application. This information is abstracted from a communication addressed to Prof. T. Gaillard Thomas, by Dr. R., and published in the New York *Medical Journal.*

**Albuminuric Retenitis of Pregnancy.**

Dr. A. Emery Jones calls attention to this condition in the *British Medical Journal.* The prognosis he considers good, so far as vision is concerned, though sometimes patches of choroidal atrophy remain which considerably diminish the acuteness of vision. For treatment he recommends iron in some form, and extra care. He hopes that this note will induce practitioners to take careful notes of the presence of albumen during pregnancy, and the effect on the labor itself, and on the general health of the patient afterwards. A combination, suggested by Dr. Bulkley, of a drachm of washed sulphur, a half fluid ounce of ether and three and a half fluid ounces of alcohol makes an excellent application, and is especially indicated in those cases in which comedones are predominant, or in which there is a moderate degree of seborrhœa present. Kummerfields' lotion is another very useful combination.

**Pulmonary Consumption.**

F. J. B. Quinlon, M. D., M. R. S. A., F. R. Q. C. P., Physician to St. Vincent Hospital, Dublin, recommends the use of verbascum thapsus, or great mullen, in hot milk decoction, in the treatment of this affection. He says (*British Medical Journal*): "That it eases phthisical cough there can be no doubt; in fact, some of the patients scarcely took their cough mixtures at all—an unmixed boon to phthisical sufferers with delicate stomachs. Its power of checking phthisical looseness of the bowels was very marked, and experiment proved that this was not due to the well-known astringent properties of boiled milk. It also gave great relief to the dyspnœa. For phthisical night sweats it is utterly useless." Like other vegetable remedies employed by the medical profession, good or bad, the use of this agent in the hands of scientific men is antedated by its employment in the hands of the unscientific. It is the prerogative of the physician to test all things, rejecting the bad and holding to that which is good. And when he finds a good thing, he owes the publication of the fact in payment of his debt to science and his profession.

**Scabies.**

Naphthalin was first employed in scabies by Kaposi, its value being suggested by the use which furriers make of it as an insecticide. It has been further used in the treatment of sixty cases of this disease by Furbringer, who reports that it is most successful. It is employed in a 10 or 20 per cent. solution in oil. In one case it caused a slight and very transient albuminuria. Although the parasitic affection was quickly cured, naphthalin seems to have no influence on the secondary eruptions, which, in some cases of irritable skin seem to be augmented by it. In other skin diseases, especially those in which large surfaces are exposed, naphthalin is to be avoided. Furbringer treated a case of psoriasis with a daily application of five to ten grammes of the above solution without benefit to the skin eruption, and with the result of causing a nephritic albuminuria. Neither in chronic eczema, nor in tinea tonsurans, did it do good.—*Lancet.*

**Tape-Worm.**

At a recent meeting of the New York Pathological Society Dr. Sell presented two specimens of tænia Solium.

One was obtained from a female patient, thirty years of age, single, who had been aware of the presence of the worm for three years, the other was obtained from a female patient, thirty years of age, married, who had been aware of the presence of the worm for three months.

The plan of treatment was somewhat different from that which he had reported at a former meeting, and was as follows: Ten grains of calomel were given on a Friday evening. On Saturday the patients took nothing into their stomachs except stewed pumpkin and slippery-elm tea. On Sunday they took the following mixture, divided into four equal portions, and administered at intervals of fifteen minutes:

Pumpkin Seeds, 1 ounce.
Ethereal extract of male fern, 1 drachm.
Kameela, 2 drachms.

If the patient has not a free evacuation from the bowels within two hours after taking the last dose, or as soon as gurgling, with or without colicky pain, appears, administer an ounce of Rochelle salts, or Epsom salts, or, in cold weather, castor oil. When purgation begins, have the patient sit over a pail partly filled with warm water.

To prepare the medicine, remove the outer envelope of one ounce of fresh pumpkin seeds, and then beat them into a paste with one hundred grains of sugar finely powdered. To this paste add half a fluid ounce or more of water, and when a homogeneous mixture has been obtained add the other ingredients gradually, and to the whole add water sufficient to make four ounces.

